# Association of Dietary Changes with Risk Factors of Type 2 Diabetes among Older Adults in Sharpeville, South Africa, from 2004 to 2014

**DOI:** 10.3390/nu15224751

**Published:** 2023-11-11

**Authors:** Hyunjung Lee, Gugulethu T. Moyo, Rufus J. Theophilus, Wilna Oldewage-Theron

**Affiliations:** 1 Department of Nutrition, Texas A&M University, College Station, TX 77843, USA; 2 Center for Health and Wellbeing, Princeton School of Public and International Affairs, Princeton University, Princeton, NJ 08544, USA; gugulethu.moyo@princeton.edu; 3 Food Science and Human Nutrition Department, University of Florida, Gainesville, FL 32611, USA; 4 Department of Nutritional Sciences, Texas Tech University, Lubbock, TX 79409, USA; 5 Department of Sustainable Food Systems and Development, University of the Free State, Bloemfontein 9301, South Africa

**Keywords:** diabetes mellitus, aging, diet, overweight, obesity, food insecurity

## Abstract

This study aimed to evaluate the associations of dietary changes with risk factors of type 2 diabetes among older populations in Sharpeville, South Africa. A 24 h recall assessment, dietary diversity, and anthropometrics were measured. Blood samples were collected to assess fasting glucose and insulin. Regression analysis was performed using SPSS version 20. The mean BMI of the total of 103 participants was 30.63 kg/m^2^ at baseline and 29.66 kg/m^2^ at follow-up. Significantly higher BMI levels were reported in women than men both at baseline (*p* = 0.003) and follow-up (*p* = 0.009). Waist circumference significantly decreased from 96.20 cm to 93.16 cm (*p* = 0.046). The mean levels of HOMA-B significantly increased from 88.99 to 111.19 (*p* = 0.021). BMI was positively associated with intakes of total energy (*p* = 0.22), polyunsaturated fatty acids (*p* = 0.050), and cholesterol (*p* = 0.006). Waist circumference was strongly associated with total energy (*p* = 0.048), polyunsaturated fatty acids (*p* = 0.037), trans fatty acids (*p* = 0.039), and cholesterol (*p* = 0.000). HOMA-IR and HOMA-B were associated with intakes of fat (HOMA-IR: *p* = 0.013; HOMA-B: *p* = 0.040) and monounsaturated fatty acids (HOMA-IR: *p* = 0.003; HOMA-B: *p* = 0.040).

## 1. Introduction

Diabetes is a rising global health crisis that disproportionately impacts low- and middle-income nations [1,2]. In 2019, global estimates indicated that 463 million adults were living with diabetes, a figure projected to swell to nearly 578 million by 2030 and possibly exceed 700 million by 2045 [1]. Among these startling numbers, the elderly are a major subset. It is forecasted that by 2045, the elderly diabetic population will surge to a worrying 276.2 million [2]. 

In Africa, it is estimated that 24 million adults have diabetes, representing every 1 in 22 adults [3]. With this terrifying incidence rate of diabetes, South Africa naturally becomes a critical locus for research because it is bedeviled by escalating diabetes cases, particularly among its elderly population—a demographic that is often under-researched. South Africa faces a severe scenario as it records the highest (12.7%) age-adjusted diabetes prevalence among African adults. This concern is magnified by a substantial diabetes-related death toll, with 89,800 fatalities registered in 2019 alone [2,3]. Alarmingly, 23.0% of the nation’s overall health spending is absorbed by diabetes, highlighting its significant financial strain [2].

Adding complexity to this health crisis is a notable gender imbalance. In South Africa, the diabetes-induced mortality rate is roughly 1.8 times higher for women compared to men [2]. This discrepancy is not merely statistical but likely stems from various influences, including biological differences and societal factors that disproportionately harm women.

Accelerating urbanization has been pivotal in escalating type 2 diabetes rates, particularly among senior citizens [4,5]. As people move from rural to urban areas, lifestyle alterations ensue. These involve shifts towards processed food consumption, reduced physical activity, sedentary behaviors, and increased stress—each linked to elevated diabetes risk [6,7]. Additional important considerations include the elderly’s higher susceptibility to severe diabetes complications like cardiovascular disorders and kidney failure [8,9]. While diet is key in managing diabetes and relevant biomarkers such as body mass index (BMI) and fasting insulin levels [10], comprehensive studies tailored to the South African context remain sparse [11,12,13,14]. Also, the subject of gender disparities is even less explored, despite distinct physiological factors affecting glucose and insulin regulation in women [15,16,17,18].

Targeted prevention and intervention measures are urgently needed for this age group. Meanwhile, the South African healthcare infrastructure is a significant factor in addressing this health emergency. However, challenges abound, from inadequate resource distribution and limited treatment avenues to poor public awareness and unequal healthcare access [19,20]. Socioeconomic inequalities, rooted in the country’s apartheid history, also have a lingering impact on dietary choices and, consequently, diabetes risk [17,21]. For example, South African women often serve as meal providers but have limited influence over food selection, contributing to poor diet [22]. Although this study is focused on South Africa, the outcomes are globally relevant. Countries experiencing similar transitions marked by urbanization and Westernization of diets—socioeconomic transitions, lifestyle changes, and health system challenges—could gain insights from this study’s findings for their diabetes management strategies [23]. The lack of such a strategy in South Africa led to an increasing trend of unhealthy eating, characterized by high sugar and low fiber intake. These dietary choices aggravate the already escalating numbers of diabetes cases, especially in marginalized populations [24].

Following this background, this study aims to contribute by closely examining the relationships between dietary changes and risk factors of diabetes, including BMI, waist circumference, and other biochemical markers such as Homeostatic Model Assessment for Insulin Resistance (HOMA-IR), and Homeostatic Model Assessment for β-Cell Function (HOMA-B) over an approximately 10-year period, from 2004 to 2014, specifically among the Sharpeville elderly community. In addition, this study aims to differentiate between gender-specific outcomes. The unique combination of physiological factors and social determinants of health in South Africa makes it imperative to conduct gender-specific research. This study not only seeks to fill an existing research gap, but also holds the potential to inform healthcare strategies and interventions, particularly for women.

## 2. Materials and Methods

### 2.1. Study Design

The cross-sectional study was conducted at baseline in 2004 and follow-up in 2014. 

### 2.2. Location

This research was conducted within an elderly care facility located in Sharpeville, situated in the Vaal region of the Gauteng Province of South Africa. Sharpeville is one of the oldest among six townships (communities) located in the Vaal region, an area characterized by industrial pollution. Sharpeville had a poverty rate of 49.3% and 43.1% in 2004 [25] and 2014 [26], respectively. The newly established Sharpeville Care of the Aged day-care center, which had been in operation for three months at that time, catered to elderly individuals who visited on Mondays and Wednesdays, offering a range of services, including skills training (such as sewing, cooking, and gardening), religious activities, and the provision of free breakfast and lunch. These services were primarily aimed at addressing the needs of food-insecure elderly (≥60 years old) individuals with limited resources, most of them caring for human immunodeficiency virus (HIV)- and acquired immunodeficiency syndrome (AIDS)-affected orphans (grandchildren). In 2004, the management of the aforementioned elderly care center initiated contact with the researchers, seeking their assistance in conducting a cross-sectional baseline survey to aid in their strategic planning efforts for improving the general wellbeing of these elderly individuals. It is important to note that this care center is the sole facility of its kind in Sharpeville.

### 2.3. Sampling

The initial phase of this study involved the recruitment of participants from the newly established Sharpeville Care of the Aged day-care center, which had been in operation for three months at that time. 

To determine an appropriate sample size for the cross-sectional survey within this elderly community in 2004, a sample size calculator was employed, and it was determined that 169 participants were required to obtain statistically representative data [27]. A random selection process was employed to choose 170 men and women from an alphabetical list of names supplied by the center management (comprising a total of 350 names) for participation in the study. No specific exclusion criteria were applied, and any individual attending the care center who provided informed consent was eligible for inclusion. To facilitate effective communication with the diverse linguistic backgrounds of the participants, eight fieldworkers were recruited and trained using a comprehensive training manual and participatory facilitating methods [27].

In 2014, a follow-up investigation was conducted by revisiting the elderly care center. It was found that only 105 out of the original 169 participants from the 2004 study were still actively attending the center on a voluntary basis and were, therefore, included in the cohort phase of the research. All 105 of these individuals willingly provided their consent to participate in the follow-up study, resulting in a follow-up rate of 63.3%. Consistency was maintained in terms of measurement procedures and the use of standardized measuring instruments for both the 2004 baseline and 2014 data collection. 

### 2.4. Measurements for Both 2004 and 2014

A structured questionnaire was used to obtain information on the age, employment status, and monthly household income of the participants [27]. A 24 h recall questionnaire was administered over a span of three non-consecutive days, encompassing one weekend day and two weekdays. In light of the provision of breakfast and lunch during care center attendance days, it was decided to include a 24 h dietary recall assessment for two days attending the care center and one day when the elderly individuals were not participating in care center activities. This approach was undertaken to obtain quantitative, descriptive information into their usual food consumption patterns and dietary intakes. Trained fieldworkers employed the four-stage, multiple-pass one-on-one interviewing procedure as outlined by Gibson [28]. In order to estimate portion sizes accurately, food models were utilized by the fieldworkers. A registered dietitian analyzed the dietary nutrient intake data using the FoodFinder^®^ version 3 software program. This software program was developed by the Medical Research Council and based on the South African food composition tables [29]. The average macro- and micronutrient intakes for the three-day period were computed for each participant.

Dietary diversity was calculated over a reference period of seven days, as recommended by Ruel [30]. The calculation of the Dietary Diversity Score (DDS) in this study adhered to the methodology used in previous research conducted in developing countries [31,32], and nine nutritious food groups were used to align with the recommendations of the United Nations Food and Agriculture Organization. The food groups included were as follows: (1) cereals, roots, and tubers; (2) dairy products; (3) eggs; (4) fats and oils; (5) legumes and nuts; (6) other vegetables; (7) other fruits; (8) vitamin A-rich fruits and vegetables; and (9) flesh products, which include meat, poultry, fish, and offal/tripe [33]. A modified and validated seven-day Food Frequency Questionnaire (FFQ) was administered to the participants. The DDS was computed across all nine food groups [30,32]. Matla described the cut-off points for a low, medium, and high DDS as 0–3 food groups, 4–5 food groups, and 6–9 food groups, respectively [33].

Weight and height measurements according to standardized procedures [34] were conducted by a registered dietitian and a public health nutritionist. Weight was measured on a Philips electronic bathroom scale model HF350, and height was measured with a Scales 2000 portable stadiometer. In adherence to standardized protocols, two measurements were taken for each parameter, with a stipulated tolerance of no more than 0.1 kg for weight and 5 mm for height between the two measurements. In the event of any variance within these limits, the average of the two measurements was employed for analysis [34]. Body mass index was calculated as weight (kg) divided by height squared (m^2^). 

Waist circumference (WC) was measured by a registered dietitian at the midpoint between the lower rib and the iliac crest using a non-stretchable measuring tape, positioned horizontally around the body [28]. A WC of >88 cm for women and >102 cm for men indicates a greater risk for chronic diseases of lifestyle [28].

During both the 2004 baseline and 2014 data collection, consistent with protocol, the same two nursing practitioners and a hematologist were responsible for drawing venous blood samples in 3 mL glucose tubes containing sodium fluoride and oxalate, following a requisite overnight fast of not more than 10 h, within the timeframe of 06h00 to 10h00. The use of tourniquets was minimized, and the collected blood specimens were promptly placed on ice to shield them from direct exposure to sunlight. Within a span of two hours from the time of collection, low-speed (3000 rpm for 30 s) centrifugation of serum and plasma was carried out at a controlled temperature of 4 °C. The serum and plasma samples were then aliquoted into individual tubes and stored at a temperature of −80 °C until analysis. Rigorous adherence to established laboratory protocols was upheld to ensure compliance with the accreditation standards set forth by the South African National Accreditation System (SANAS) and overseen by the hematologist.

The Konelab 20i random access automated clinical chemistry system was employed for the analysis of serum glucose levels. The coefficient of variation (percent CV) between runs fell within the range of 1.2–2.8%, indicating the precision of the measurements. The Konelab 20i relies on colorimetric and turbidimetric measuring principles. Serum glucose level reference values of 4.1–5.9 mmol/L were used [35]. A sandwich immunoluminometric assay on an automated Maglumi 1000 system was used to determine the serum insulin level. The precision inter- and intra-assay coefficient of variance was ≤15% [36]. Reference values used were 4.03–23.46 μIU/mL [36]. HOMA-IR was calculated using the following formula: fasting insulin (μIU/mL) × fasting glucose (mmol/mL)/22.5. HOMA-B was calculated using the following formula: 20 × fasting insulin (μIU/mL)/fasting glucose (mmol/mL) − 3.5 [10]. 

### 2.5. Statistical Analysis

A complete dataset of 103 participants was obtained and used for statistical analyses. The following variables were extracted from the database for each participant in order to carry out the analysis: age, BMI, waist circumference, glucose, insulin, HOMA-IR, and HOMA-B for men and women in 2004 and 2014 as the dependent variables. The dietary assessment framework included the following 11 independent variables: dietary diversity, total energy, dietary protein, fat percentage, saturated fatty acid percentage, monounsaturated fatty acid percentage, polyunsaturated fatty acid percentage, trans fatty acid percentage, dietary cholesterol, carbohydrates, sugar percentage, and fiber percentage. For this study, the analysis only considered those with a complete set of information at each of the two time points in 2004 and 2014. 

The data extracted were processed using SPSS version 20. The data were analyzed using descriptive and inferential statistics procedures. Descriptive statistics were used to assess the central tendency and distribution of the study variables (mean scores, standard deviation, median, and frequency). The sample t-test was used to compare means between men and women at the given two time points. The Pearson chi-square independence test was used to evaluate the association of categorical variables. For the comparison between mean values for men, women, and total participants for the two time points, analysis of variance (ANOVA) was performed. To differentiate between the different tests, *p* * was used to denote the *t*-test, and *p* ** represented ANOVA. Multivariate linear regression analyses were performed to model individual relationships with all the potential predictor dietary variables. The variables with a *p* value less than 0.05 in their respective univariate models were then combined to fit simultaneously in a multiple logistic model. Age and sex were adjusted in all regression models.

## 3. Results

### 3.1. Background Characteristics of Study Participants

Among the 103 participants included in the analyses, 87 (84.5%) were females and 16 (15.5%) were males. The mean age of the total participants was 68.01 in 2004 and 78.02 in 2014. By gender, the mean age was 68.28 for females and 66.56 for males in 2004 and 78.29 for females and 76.56 for males in 2014 (Table 1). The mean BMI was 30.63 kg/m^2^ in 2004 and 29.66 kg/m^2^ in 2014. A significant difference between the mean score for BMI by gender was observed: women had significantly higher BMI levels than men both in 2004 (*p* = 0.003) and 2014 (*p* = 0.009). The mean scores of waist circumference and HOMA-B differ significantly between the two time points. The average waist circumference of the total participants significantly decreased from 96.20 cm in 2004 to 93.16 cm in 2014 (*p* = 0.046). The mean scores of HOMA-B of the total participants significantly increased from 88.99 in 2004 to 111.19 in 2014 (*p* = 0.021). The mean fasting glucose levels of the total participants were 6.07 mmol/L in 2004 and 5.90 mmol/L in 2014. The mean fasting insulin levels were 26.45 uIU/mL in 2004 and 30.55 uIU/mL in 2014. No significant differences in mean scores were observed in other characteristics, such as age, glucose, insulin, and HOMA-IR by the genders and two time points.

Most (> 70%) of the participants did not meet the recommended guidelines for BMI, waist circumference, and HOMA-B in 2004 and 2014 (Table 2). More than half (>50%) of the participants had abnormal values for insulin and HOMA-IR at the two time points. Of all participants, 48.5% and 44.7% had abnormal values for fasting glucose levels in 2004 and 2014. A non-parametric Pearson chi-square test did not reveal any statistically significant associations between gender, time points, and different diabetes indicators. This, however, should not be construed to imply an absence of a relationship or a particular trend.

### 3.2. Comparision of Dietary Diversity and Nutrients

The median scores and IQR values were computed and compared to the WHO dietary range. The dietary diversity score median was 7 with an IQR of 0–8 (interquartile range 25–75th percentile) for the base period in 2004 and 8 with an IQR of 8–9 for the follow-up in 2014, and these are within the recommended DRI bracket (Table 3). On average, women consumed a total energy of 5425.3 kJ/day in 2004 and 5159.4 kJ/day in 2014, which is below the recommended amount. Similarly, men reported inadequate intake of total energy in 2004 (6024.8 kcal/day) and 2014 (4917.8 kcal/day). The increment in BMI despite lower caloric intake could have occurred for several reasons, some of which include the following: (i) Self-reported data might be subject to underreporting. (ii) Participants’ activity levels (not assessed in this study) are more likely to decline with age [37]. This might result in weight gain even with low caloric intake. In other words, with the increased likelihood of a sedentary lifestyle at this age, the balance between energy intake and expenditure might favor a BMI increase. (iii) As participants are different, the metabolic rates/changes occurring in the participants are different [38]. (iv) Diet quality might also be a factor, if the nutrient density is reduced over the years, as consuming fatty foods and foods high in sugar but low in other nutrients might result in weight gain [39]. (v) Also, since no strict exclusion criteria were enforced in this study, some participants may have developed health conditions that affected their weight irrespective of caloric intake [40].

For total participants, intakes of monosaturated fatty acids, polyunsaturated fatty acids, and fiber were below the recommended guidelines, while high protein and carbohydrate intakes, above the recommended amount, were observed at the two time points.

### 3.3. Multivariate Linear Regression Analysis

Univariate linear regression analyses were performed to model individual relationships with all the potential predictor dietary variables. There were statistically significant associations between BMI levels and total energy intake (95% CI: 0.000, 0.001; *p* = 0.022). An increase in BMI in older adults was significantly associated with polyunsaturated fatty acid intake (95% CI: 0.001, 1.973; *p* = 0.050). Increased intake of cholesterol was associated with an increase in BMI (95% CI: 0.026, 0.148; *p* = 0.006). There were no statistically significant associations of BMI with dietary diversity and intakes of carbohydrates, protein, fat, saturated fatty acids, monounsaturated fatty acids, sugar, and fiber (Table 4).

Waist circumference was significantly associated with intakes of total energy (95% CI: 0.000, 0.002; *p* = 0.048), polyunsaturated fatty acids (95% CI: 0.133, 4.154; *p* = 0.037), trans fatty acids (95% CI: −0.030, −0.001; *p* = 0.039), and cholesterol (95% CI: 0.110, 0.360, *p* = 0.000). Waist circumference was found to have no significant associations with dietary diversity and intakes of carbohydrates, protein, fat, saturated fatty acids, monounsaturated fatty acids, sugar, and fiber (Table 5).

Increased intake of total fat had a strong positive association with high fasting insulin levels (95% CI: 0.065, 11.073; *p* = 0.047). The intake of monounsaturated fatty acids was also significantly associated with fasting insulin levels (95% CI: 0.330, 10.238, *p* = 0.037). There were no significant associations of fasting insulin levels with dietary diversity and other nutrient intakes such as carbohydrates, protein, saturated fatty acids, polyunsaturated fatty acids, trans fatty acids, cholesterol, sugar, and fiber (Table 6).

Fasting glucose levels were not strongly associated with dietary diversity and other dietary intakes (Table 7).

The HOMA of Insulin Resistance (HOMA-IR) index had a significantly positive association with fat intake (95% CI: 0.421, 3.561; *p* = 0.013). Increased intakes of monounsaturated fatty acids (95% CI: 0.716, 3.542; *p* = 0.003) and cholesterol (95% CI: 0.015, 0.227; *p* = 0.026) were associated with a high HOMA index. HOMA index was found to have no significant associations with dietary diversity and intakes of total energy, carbohydrates, protein, saturated fatty acids, polyunsaturated fatty acids, trans fatty acids, sugar, and fiber (Table 8).

The HOMA of β-Cell Function (HOMA-B) index had significantly positive associations with fat intake (95% CI: 1.018, 43.539; *p* = 0.040) and monounsaturated fatty acids (95% CI: 0.915, 39.223; *p* = 0.040). HOMA-B had no statistically significant associations with dietary diversity and intakes of total energy, carbohydrates, protein, saturated fatty acids, polyunsaturated fatty acids, trans fatty acids, cholesterol, sugar, and fiber (Table 9).

The variables with *p* value less than 0.05 in the univariate models were selected and combined to fit simultaneously in a multiple logistic model. The independent dietary variables of total energy, carbohydrates, cholesterol levels, polyunsaturated fatty acid percentage, saturated fatty acid percentage, and trans fatty acid percentage were simultaneously computed in the final predictor model. The assumption was that each independent variable has a somewhat linear relation with waist circumference (dependent variable) and is a good indicator for risk factors of type 2 diabetes. The results of the multiple linear regression analyses show that intakes of carbohydrates (95% CI: 0.138, 0.372; *p* = 0.000), polyunsaturated fatty acids (95% CI: 0.333, 1.613; *p* = 0.003), and cholesterol (95% CI: −0.029, −0.004; *p* = 0.011) had statistically significant associations with waist circumference (Table 10).

## 4. Discussion

The present cross-sectional study investigated the association of dietary changes with risk factors of type 2 diabetes in older adults living in Sharpeville, South Africa. A majority of the study subjects reported abnormal levels of BMI, waist circumference, fasting insulin, HOMA-IR, and HOMA-B in 2004 and 2014. In addition, high carbohydrate intakes were observed in both men and women at baseline and follow-up. Intakes of monounsaturated fatty acids, polyunsaturated fatty acids, and fiber were constantly lower than their respective recommended ranges at both time points.

Most of the participants in this study were overweight. The results of univariate regression analysis showed a positive association of BMI with intakes of polyunsaturated fatty acids. In contrast to our findings, a cross-sectional study conducted among adults 19 years and older found that BMI was not associated with the intake of polyunsaturated fatty acids [41]. A previous prospective study also found a positive association between weight reduction and increasing intakes of polyunsaturated fatty acids at the expense of saturated fatty acids [42]. A balanced dietary omega-6 to omega-3 fatty acid ratio is very important in the prevention and management of obesity. In a recent study of a UK national diet and nutritional survey, a high ratio of dietary omega-6/omega-3 fatty acid intakes was positively associated with obesity risk [43]. Similarly, a prospective study of middle-aged women showed that a high omega-6 to omega-3 ratio was associated with a risk of becoming overweight or obese [44]. The use of diets low in omega-6/omega-3 ratio could be an effective approach to weight management for adults with risk factors of diabetes, such as a high BMI.

This study found that increased waist circumference was strongly associated with the percentage of calories from fat intake, such as trans fatty acids. Aligning with our findings, in other prospective cohort studies, intakes of trans fatty acids have been positively associated with increases in waist circumference [45]. The results of a previous randomized controlled study also showed that intakes of trans fatty acids increased waist circumference [46]. However, the effects of trans fatty acids on weight remain controversial. An intake of artificial trans fatty acids (manufactured by partial hydrogenation of vegetable oils and containing unsaturated fatty acids) of 0.7 g/day was positively associated with obesity induction [47]. The same study also found natural fatty acids (containing unsaturated fatty acids and coming from ruminant animals) had no association [47].

In addition, the results of multivariate analyses in this study showed that increased waist circumference was strongly associated with the percentage of calories from carbohydrates. Findings of a recent randomized controlled study suggested that a low-carbohydrate diet (below 26% of total energy) led to a significant reduction in waist circumference among adults with overweight or obesity [48]. Bazzano et al. [49] also found that diets low in carbohydrates (<40 g/day) resulted in favorable changes in waist circumference. In addition, a balanced ratio of the composition of polyunsaturated fatty acids, particularly the omega-6/omega-3 ratio, has been an important point of discussion in managing body weight. Studies have shown that an increased ratio of omega-6:omega-3 fatty acids in a diet was associated with higher waist circumference, weight, and BMI [43,50,51]. Interestingly, this study found a positive association of waist circumference with dietary intake of polyunsaturated fatty acids. A recent study conducted among the elderly in Sharpeville reported that more than 60% of older adults in this community experienced food insecurity [52]. Food insecurity is an underlying risk factor for acute and chronic malnutrition [53]. It is possible that many elderly from food-insecure households in South Africa may have insufficient intakes of omega-3 fatty acids [54]. Further research is needed to examine the current dietary status of omega-3 and omega-6 fatty acids among the elderly living in resource-poor communities.

The HOMA-IR index was positively associated with intakes of monounsaturated fatty acids and cholesterol. According to the American Diabetes Association, it is recommended that foods high in saturated fatty acids be replaced with foods rich in monounsaturated fatty acids and polyunsaturated fatty acids for diabetes prevention [55]. In contrast to our findings, monounsaturated fatty acid intake is shown to improve HOMA-IR and glucose metabolism [56]. It is possible that the associations between monounsaturated fatty acids and insulin resistance and susceptibility to developing diabetes may differ by race/ethnicity [57,58]. Evidence showing the association between dietary cholesterol and diabetes remains inconsistent. Baghdasarian et al. [59] found that there was no statistically significant association of high dietary cholesterol intakes with fasting glucose levels and the risk of type 2 diabetes among middle-aged adults (35–64 years) during 20 years of follow-up. In contrast, some animal studies showed that a high-cholesterol diet resulted in increases in body weight and fasting glucose levels and changes in insulin sensitivity [60,61].

This study has several strengths. Both baseline characteristics and inferential statistical methods were incorporated to provide a comprehensive and nuanced view of risk factors of diabetes in Sharpeville, South Africa, as it relates to long-term dietary changes, which has been lacking in previous studies. Although the scope of this study may seem broad, it does not cover other related but separate factors such as the role of physical activity and the impact of specific comorbidities (e.g., CVD, renal diseases, neuropathy, retinopathy, and obesity) on risk factors of diabetes. Moreover, this research is foundational for future investigations and invaluable as a guidepost for healthcare policy interventions both within South Africa and for the global community. A limitation of this present study is that it is impossible to draw causal relationships because of the nature of a cross-sectional study. Other valid anthropometric markers, such as calf circumference, mid-upper arm circumference (MUAC), and skinfold thickness, were not included in this study. However, since there were no significant differences in height by gender and time period in this study, BMI may serve as a proxy measure to detect overweight and obesity. Owing to the nature of this study, as opposed to a longitudinal study, the eating/dietary habits of the study populations were not investigated. An insight into these habits may further elucidate the observed increase in BMI in 2014 despite the reduced total energy intake recorded. In addition, the data collection relied on self-report methods, which may have caused recall bias. The results of this study also may be limited to generalization to other populations. Another limitation is that the last data were collected almost 10 years ago; however, another 10-year data collection is planned for 2024.

## 5. Conclusions

In conclusion, older adults of these food-insecure communities reported inadequate intakes of total energy, carbohydrates, monounsaturated fatty acids, polyunsaturated fatty acids, and fiber during a 10-year follow-up from 2004 to 2014. In this population, waist circumference values significantly increased from baseline to follow-up. High intakes of fat and cholesterol were a strong risk factor for type 2 diabetes, determined by waist circumference and HOMA-IR. Further research is needed to develop effective dietary intervention strategies to promote balanced dietary intakes of omega-6 and omega-3 fatty acids for preventing and managing type 2 diabetes among the elderly from food-insecure households in South Africa.

## Figures and Tables

**Table 1:** Baseline characteristics of the study population (*n* = 103).

Characteristic Mean (SD)	Healthy Range	2004			2014			2004		2014	
		Female	Male	Total	Female	Male	Total	*p* *	*p* **	*p* *	*p* **
Age, years		68.28 (5.98)	66.56 (4.03)	68.01 (5.74)	78.29 (6.04)	76.56 (4.03)	78.02 (5.79)	0.184	0.274	0.174	0.276
Height (cm)		156.61 (0.10)	156.71 (0.09)	156.71 (0.09)	157.00 (0.09)	157.20 (0.09)	157.20 (0.09)	0.298	0.238	0.249	0.238
BMI (kg/m^2^)	18.5 to 24.9	31.40 (6.02)	26.45 (5.49)	30.63 (6.19)	30.29 (5.69)	26.20 (5.22)	29.66 (5.79)	0.498	0.003	0.385	0.009
Waist circumference (cm)	Female >80/ Male >90	96.66 (10.97)	94.20 (14.77)	96.27 (11.60)	93.11 (11.31)	93.38 (14.16)	93.16 (11.73)	0.046	0.441	0.129	0.935
Glucose (mmol/L)	3.9–5.6	6.05 (3.01)	6.19 (1.98)	6.07 (2.86)	6.08 (2.52)	4.90 (1.19)	5.90 (2.40)	0.531	0.852	0.081	0.07
Insulin (μIU/mL)	4.03–23.46	27.76 (32.96)	19.19 (21.06)	26.45 (31.50)	31.15 (31.66)	27.29 (26.51)	30.55 (30.83)	0.27	0.335	0.515	0.648
HOMA-IR	<2	7.99 (10.45)	4.95 (7.12)	7.52 (10.05)	8.04 (8.40)	7.02 (6.34)	7.88 (8.10)	0.199	0.282	0.522	0.646
HOMA-β	<1.9	96.79 (120.01)	45.29 (37.65)	88.99 (112.90)	109.56 (128.80)	120.03 (114.08)	111.19 (126.16)	0.021	0.104	0.99	0.762

Note: *p* *: *t*-test, *p* **: ANOVA.

**Table 2:** Baseline characteristics of the study population.

Variable	2004 (% of Abnormal Values)			2014 (% of Abnormal Values)		
	Female	Male	Total	Female	Male	Total
BMI	82.8	68.8	80.6	79.3	68.8	77.7
Waist circumference	93.1	75.0	90.3	83.9	56.2	79.6
Glucose	48.3	50.0	48.5	40.2	68.8	44.7
Insulin	56.3	25.0	51.5	62.1	43.8	59.2
HOMA-IR	65.5	56.2	64.1	77.0	81.2	77.7
HOMA-β	94.3	93.8	94.2	98.9	100	99.0

**Table 3:** Comparison of diet adequacy between 2004 and 2014.

Variable	Full				Women				Men				DRI/ WHO
	2004 Mdn	2004 IQR	2014 Mdn	2014 IQR	2004 Mdn	2004 IQR	2014 Mdn	2014 IQR	2004 Mdn	2004 IQR	2014 Mdn	2014 IQR	
Dietary Diversity Score	7.0	0.0–8.0	8.0	8.0–9.0	7.0	0.0–8.0	8.0	8.0–9.0	7.0	0.0–9.0	9.0	7.0–9.0	0.0–9.0
TE (kj)	5472.0	3951.0–7242.0	5151.0	3733.0–6638.0	5425.3	3871.0–7030.0	5159.4	3729.0–6643.0	6024.8	4206.0–8819.0	4917.8	4263.0–6643.0	9942.0 (m) 6362.0 (w)
Carbohydrates (g)	147.4	70.0–217.6	134.0	64.0–233.7	141.6	85.5–202.4	133.8	89.4–169.0	130.0	87.4–219.3	129.8	87.3–162.6	100
Protein (g)	61.0	39.0–85.0	62.0	39.0–89.0	59.3	39.0–81.0	65.8	40.0–88.0	68.5	39.0–106.0	48.8	31.0–103.0	46.0
Total Fat (%)	27.0	19.0–34.0	26.0	20.0–36.0	27.2	19.0–34.0	26.1	20.0–36.0	24.8	20.0–36.0	25.7	18.0–39.0	<30.0% TE
Saturated fatty acids (%)	8.0	5.0–13.0	8.0	6.0–13.0	8.3	5.0–13.0	8.3	6.0–12.0	9.2	6.0–13.0	9.3	7.0–14.0	<10.0% TE
MUFAs (%)	10.0	6.0–14.0	10.0	6.0–14.0	10.2	6.0–14.0	10.2	6.0–13.0	9.0	6.0–16.0	9.6	6.0–16.0	15.0–20.0%
PUFAs (%)	5.0	3.0–7.0	5.0	3.0–6.0	5.0	3.0–7.0	5.0	3.0–6.0	5.0	3.0–6.0	4.0	2.0–7.0	6.0–11.0% TE
TFAs (%)	0.0	0.0–1.0	0.0	0.0–1.0	0.3	0.0–1.0	0.2	0.0–1.0	0.2	0.0–1.0	0.6	0.0–1.0	<1.0% TE
Cholesterol (mg)	108.0	52.0–256.0	159.0	83.0–273.0	111.7	47.0–231.0	167.6	85.0–268.0	83.8	56.0–294.0	114.1	77.0–365.0	<300.0
Added Sugar (%)	4.0	2.0–6.0	5.0	3.0–7.0	3.6	2.0–6.0	4.7	3.0–7.0	2.9	2.0–6.0	4.9	2.0–8.0	<10.0%
Fiber (g)	3.0	2.0–5.0	3.0	2.0–5.0	3.1	2.0–5.0	3.2	2.0–5.0	3.2	2.0–5.0	3.1	2.0–5.0	30.0 (m) 21.0 (w)

Abbreviations: g: gram; kj: kilojoule; m: men; MUFAs: monounsaturated fatty acids; PUFAs: polyunsaturated fatty acids; TFAs: trans fatty acids; TE: total energy; w: women.

**Table 4:** Regression coefficients for predicting body mass index.

Variable	Unstandardized Coefficient		Standardized Coefficient			95% Confidence Interval for B	
	B	Std. Error	Beta	*t*	*p*	Lower Bound	Upper Bound
(Constant)	25.437	5.427		4.687	0.000	14.731	36.144
Gender	4.390	1.097	0.266	4.001	0.000	2.226	6.554
Age	−0.099	0.056	−0.127	−1.759	0.080	−0.210	0.012
Dietary diversity score	0.078	0.111	0.049	0.706	0.481	−0.140	0.296
Total energy (kilojoule/day)	0.001	0.000	0.246	2.316	0.022	0.000	0.001
Carbohydrate percentage	−0.019	0.019	−0.116	−1.019	0.309	−0.057	0.018
Dietary protein (gram/day)	−0.169	0.345	−0.370	−0.488	0.626	−0.850	0.513
Fat percentage	0.018	0.484	0.018	0.038	0.970	−0.937	0.974
Saturated fatty acid percentage	0.533	0.371	0.531	1.435	0.153	−0.200	1.265
MUFA percentage	0.289	0.437	0.133	0.662	0.509	−0.573	1.151
PUFA percentage	0.987	0.500	0.171	1.975	0.050	0.001	1.973
TFA percentage	−0.003	0.004	−0.069	−0.807	0.420	−0.010	0.004
Cholesterol (mmol/L)	0.087	0.031	0.282	2.803	0.006	0.026	0.148
Sugar percentage	−0.029	0.023	−0.090	−1.245	0.215	−0.074	0.017
Fiber percentage	−0.167	0.190	−0.067	−0.879	0.380	−0.543	0.208

Note: R square (adj) = 0.177; R square shows that 17.7% of the variation in BMI is explained by the model (*n* = 203, *p* = 0.00); F statistic = 0.00, the significance value of the F statistic is less than 0.05, which means that the variation explained by the model is not due to chance. Abbreviations: MUFA: monounsaturated fatty acid; PUFA: polyunsaturated fatty acid; TFA: trans fatty acid.

**Table 5:** Regression coefficients for predicting waist circumference.

Variable	Unstandardized Coefficient		Standardized Coefficient			95% Confidence Interval for B	
	B	Std. Error	Beta	*t*	*p*	Lower Bound	Upper Bound
(Constant)	79.003	11.167		7.075	0.000	56.971	101.036
Gender	−0.747	2.228	−0.023	−0.335	0.738	−5.143	3.649
Age	−0.047	0.117	−0.030	−0.403	0.687	−0.278	0.183
Dietary diversity score	0.320	0.227	0.102	1.409	0.161	−0.128	0.768
Total energy (kilojoule/day)	0.001	0.001	0.218	1.988	0.048	0.000	0.002
Carbohydrate percentage	−0.061	0.039	−0.183	−1.557	0.121	−0.137	0.016
Dietary protein (gram/day)	−0.473	0.703	−0.533	−0.672	0.502	−1.860	0.915
Fat percentage	0.617	0.986	0.319	0.626	0.532	−1.328	2.562
Saturated fatty acid percentage	0.704	0.755	0.361	0.932	0.353	−0.787	2.194
MUFA percentage	1.377	0.887	0.322	1.553	0.122	−0.373	3.127
PUFA percentage	2.144	1.019	0.191	2.104	0.037	0.133	4.154
TFA percentage	−0.015	0.007	−0.186	−2.076	0.039	−0.030	−0.001
Cholesterol (mmol/L)	0.235	0.063	0.387	3.704	0.000	0.110	0.360
Sugar percentage	−0.055	0.047	−0.088	−1.170	0.244	−0.148	0.038
Fiber percentage	−0.010	0.388	−0.002	−0.026	0.979	−0.775	0.755

Note: R square (adj) = 0.161; R square shows that 16.1% of the variation in waist circumference is explained by the model. Abbreviations: MUFA: monounsaturated fatty acid; PUFA: polyunsaturated fatty acid; TFA: trans fatty acid.

**Table 6:** Regression coefficients for predicting fasting insulin level.

Variable	Unstandardized Coefficient		Standardized Coefficient			95% Confidence Interval for B	
	B	Std. Error	Beta	*t*	*p*	Lower Bound	Upper Bound
(Constant)	16.843	32.038		0.526	0.600	−46.369	80.054
Gender	3.598	6.383	0.041	0.564	0.574	−8.996	16.191
Age	−0.235	0.326	−0.058	−0.720	0.472	−0.878	0.408
Dietary diversity score	0.432	0.655	0.051	0.661	0.510	−0.859	1.724
Total energy (kilojoule/day)	−0.001	0.001	−0.051	−0.432	0.666	−0.003	0.002
Carbohydrate percentage	−0.023	0.112	−0.026	−0.203	0.839	−0.243	0.197
Dietary protein (gram/day)	−1.951	2.001	−0.822	−0.975	0.331	−5.899	1.996
Fat percentage	5.569	2.790	1.076	1.996	0.047	0.065	11.073
Saturated fatty acid percentage	−0.927	2.157	−0.178	−0.430	0.668	−5.182	3.328
MUFA percentage	5.284	2.511	0.465	2.104	0.037	0.330	10.238
PUFA percentage	0.259	2.883	0.009	0.090	0.928	−5.429	5.947
TFA percentage	−0.008	0.021	−0.035	−0.366	0.715	−0.049	0.034
Cholesterol (mmol/L)	0.319	0.189	0.197	1.691	0.093	−0.053	0.692
Sugar percentage	−0.108	0.133	−0.065	−0.810	0.419	−0.371	0.155
Fiber percentage	−0.234	1.136	−0.018	−0.206	0.837	−2.476	2.008

Note: R square (adj) = 0.015; R square shows that only 0.15% of the variation in fasting insulin levels is explained by the model. Abbreviations: MUFA: monounsaturated fatty acid; PUFA: polyunsaturated fatty acid; TFA: trans fatty acid.

**Table 7:** Regression coefficients for predicting fasting glucose level.

Variable	Unstandardized Coefficient		Standardized Coefficient			95% Confidence Interval for B	
	B	Std. Error	Beta	*t*	*p*	Lower Bound	Upper Bound
(Constant)	4.196	2.712		1.548	0.123	−1.153	9.546
Gender	0.412	0.547	0.056	0.753	0.452	−0.667	1.492
Age	0.006	0.028	0.019	0.230	0.818	−0.049	0.062
Dietary diversity score	−0.035	0.056	−0.050	−0.630	0.529	−0.145	0.075
Total energy (kilojoule/day)	0.000	0.000	−0.037	−0.309	0.758	0.000	0.000
Carbohydrate percentage	−0.002	0.010	−0.025	−0.198	0.843	−0.021	0.017
Dietary protein (gram/day)	−0.011	0.172	−0.056	−0.066	0.948	−0.351	0.329
Fat percentage	−0.054	0.242	−0.124	−0.225	0.822	−0.531	0.422
Saturated fatty acid percentage	0.124	0.185	0.280	0.671	0.503	−0.241	0.489
MUFA percentage	0.005	0.218	0.005	0.022	0.982	−0.425	0.434
PUFA percentage	0.109	0.249	0.043	0.437	0.663	−0.383	0.601
TFA percentage	0.001	0.002	0.072	0.743	0.458	−0.002	0.005
Cholesterol (mmol/L)	0.016	0.015	0.119	1.043	0.298	−0.014	0.047
Sugar percentage	−0.007	0.012	−0.051	−0.622	0.535	−0.030	0.016
Fiber percentage	0.013	0.095	0.012	0.134	0.894	−0.175	0.201

Note: R square (adj) = 0.034; R square shows that only 3.4% of the variation in fasting glucose levels is explained by the model. Abbreviations: MUFA: monounsaturated fatty acid; PUFA: polyunsaturated fatty acid; TFA: trans fatty acid.

**Table 8:** Regression coefficients for predicting HOMA-IR.

Variable	Unstandardized Coefficient		Standardized Coefficient			95% Confidence Interval for B	
	B	Std. Error	Beta	*t*	*p*	Lower Bound	Upper Bound
(Constant)	−2.688	9.140		−0.294	0.769	−20.722	15.345
Gender	0.815	1.821	0.032	0.447	0.655	−2.778	4.407
Age	−0.018	0.093	−0.015	−0.197	0.844	−0.202	0.165
Dietary diversity score	−0.040	0.187	−0.016	−0.213	0.831	−0.408	0.329
Total energy (kilojoule/day)	0.000	0.000	−0.074	−0.644	0.520	−0.001	0.001
Carbohydrate percentage	0.011	0.032	0.043	0.345	0.730	−0.052	0.074
Dietary protein (gram/day)	−0.669	0.571	−0.966	−1.171	0.243	−1.795	0.458
Fat percentage	1.991	0.796	1.320	2.501	0.013	0.421	3.561
Saturated fatty acid percentage	−0.395	0.615	−0.259	−0.641	0.522	−1.609	0.819
MUFA percentage	2.129	0.716	0.642	2.972	0.003	0.716	3.542
PUFA percentage	0.565	0.822	0.065	0.687	0.493	−1.058	2.187
TFA percentage	−0.005	0.006	−0.078	−0.829	0.408	−0.017	0.007
Cholesterol (mmol/L)	0.121	0.054	0.256	2.247	0.026	0.015	0.227
Sugar percentage	−0.043	0.038	−0.089	−1.136	0.258	−0.118	0.032
Fiber percentage	0.026	0.324	0.007	0.081	0.936	−0.613	0.666

Note: R square (adj) =0.056; R square shows that 0.56% of the variation in HOMA-IR is explained by the model. Abbreviations: MUFA: monounsaturated fatty acid; PUFA: polyunsaturated fatty acid; TFA: trans fatty acid.

**Table 9:** Regression coefficients for predicting HOMA-β.

Variable	Unstandardized Coefficient		Standardized Coefficient			95% Confidence Interval for B	
	B	Std. Error	Beta	*t*	*p*	Lower Bound	Upper Bound
(Constant)	80.419	123.768		0.650	0.517	−163.767	324.605
Gender	9.238	24.662	0.027	0.375	0.708	−39.420	57.895
Age	−0.831	1.259	−0.053	−0.660	0.510	−3.315	1.653
Dietary diversity score	1.359	2.518	0.042	0.540	0.590	−3.608	6.327
Total energy (kilojoule/day)	0.002	0.006	0.050	0.431	0.667	−0.008	0.013
Carbohydrate percentage	−0.389	0.431	−0.114	−0.903	0.368	−1.240	0.461
Dietary protein (gram/day)	−9.873	7.731	−1.076	−1.277	0.203	−25.126	5.380
Fat percentage	22.278	10.776	1.115	2.067	0.040	1.018	43.539
Saturated fatty acid percentage	0.141	8.337	0.007	0.017	0.986	−16.307	16.590
MUFA percentage	20.069	9.708	0.459	2.067	0.040	0.915	39.223
PUFA percentage	−3.368	11.126	−0.029	−0.303	0.762	−25.318	18.583
TFA percentage	−0.061	0.081	−0.073	−0.757	0.450	−0.220	0.098
Cholesterol (mmol/L)	1.097	0.724	0.176	1.516	0.131	−0.331	2.526
Sugar percentage	−0.293	0.515	−0.046	−0.568	0.571	−1.309	0.724
Fiber percentage	−0.297	4.378	−0.006	−0.068	0.946	−8.935	8.341

Note: R square (adj) = 0.009 R square shows that only 0.9% of the variation in HOMA-B is explained by the model. Abbreviations: MUFA: monounsaturated fatty acid; PUFA: polyunsaturated fatty acid; TFA: trans fatty acid.

**Table 10:** Regression coefficients for predicting diabetes.

Dependent Variable: Waist Circumference	Unstandardized Coefficient		Standardized Coefficient			95% Confidence Interval for B	
	B	Std. Error	Beta	*t*	*p*	Lower Bound	Upper Bound
(Constant)	74.073	10.272		7.211	0.000	53.814	94.333
Gender	−0.304	2.177	−0.009	−0.140	0.889	−4.597	3.989
Age	0.004	0.110	0.003	0.038	0.970	−0.212	0.221
Total energy (kilojoule/day)	0.000	0.000	0.084	1.105	0.270	0.000	0.001
Carbohydrate percentage	0.255	0.059	0.417	4.307	0.000	0.138	0.372
Saturated fatty acid percentage	0.352	0.207	0.181	1.699	0.091	−0.057	0.760
PUFA percentage	0.973	0.324	0.230	2.998	0.003	0.333	1.613
TFA percentage	1.713	0.924	0.158	1.855	0.065	−0.108	3.535
Cholesterol (mmol/L)	−0.016	0.006	−0.202	−2.584	0.011	−0.029	−0.004

Note: R square (adj) = 0.109; R square shows that 10.9% of the variation in waist circumference is explained by the model; F statistic = 0.00, the significance value of the F statistic is less than 0.05, which means that the variation explained by the model is not due to chance. Abbreviations: MUFA: monounsaturated fatty acid; PUFA: polyunsaturated fatty acid; TFA: trans fatty acid.

## Data Availability

Data are contained within the article.

## References

[1] Saeedi P., Petersohn I., Salpea P., Malanda B., Karuranga S., Unwin N., Colagiuri S., Guariguata L., Motala A.A., Ogurtsova K. (2019). Global and regional diabetes prevalence estimates for 2019 and projections for 2030 and 2045: Results from the International Diabetes Federation Diabetes Atlas. Diabetes Res. Clin. Pract..

[2] Atlas I. (2019). IDF Diabetes Atlas Ninth.

[3] Magliano D., Boyko E. (2021). IDF Diabetes Atlas.

[4] Kahn K., Tollman S., Thorogood M., Connor M., Garenne M., Collinson M., Hundt G. (2006). Older Adults and the Health Transition in Agincourt, Rural South Africa: New Understanding, Growing Complexity.

[5] Nnam N.M. (2015). Improving maternal nutrition for better pregnancy outcomes. Proc. Nutr. Soc..

[6] Kurniawan F., Manurung M.D., Harbuwono D.S., Yunir E., Tsonaka R., Pradnjaparamita T., Vidiawati D., Anggunadi A., Soewondo P., Yazdanbakhsh M. (2022). Urbanization and Unfavorable Changes in Metabolic Profiles: A Prospective Cohort Study of Indonesian Young Adults. Nutrients.

[7] Dendup T., Feng X., Clingan S., Astell-Burt T. (2018). Environmental risk factors for developing type 2 diabetes mellitus: A systematic review. Int. J. Environ. Res. Public Health.

[8] Kirkman M.S., Briscoe V.J., Clark N., Florez H., Haas L.B., Halter J.B., Huang E.S., Korytkowski M.T., Munshi M.N., Odegard P.S. (2012). Diabetes in Older Adults. Diabetes Care.

[9] Peer N., Kengne A.-P., Motala A.A., Mbanya J.C. (2014). Diabetes in the Africa region: An update. Diabetes Res. Clin. Pract..

[10] Song Y., Manson J.E., Tinker L., Howard B.V., Kuller L.H., Nathan L., Rifai N., Liu S. (2007). Insulin sensitivity and insulin secretion determined by homeostasis model assessment and risk of diabetes in a multiethnic cohort of women: The Women’s Health Initiative Observational Study. Diabetes Care.

[11] Ley S.H., Hamdy O., Mohan V., Hu F.B. (2014). Prevention and management of type 2 diabetes: Dietary components and nutritional strategies. Lancet.

[12] Mozaffarian D. (2016). Dietary and policy priorities for cardiovascular disease, diabetes, and obesity: A comprehensive review. Circulation.

[13] Goedecke J.H., Mendham A.E. (2022). Pathophysiology of type 2 diabetes in sub-Saharan Africans. Diabetologia.

[14] Mchiza Z.J., Steyn N.P., Hill J., Kruger A., Schönfeldt H., Nel J., Wentzel-Viljoen E. (2015). A review of dietary surveys in the adult South African population from 2000 to 2015. Nutrients.

[15] Manrique-Acevedo C., Chinnakotla B., Padilla J., Martinez-Lemus L.A., Gozal D. (2020). Obesity and cardiovascular disease in women. Int. J. Obes..

[16] Kruger H.S., Puoane T., Senekal M., Van Der Merwe M.-T. (2005). Obesity in South Africa: Challenges for government and health professionals. Public Health Nutr..

[17] Puoane T., Steyn K., Bradshaw D., Laubscher R., Fourie J., Lambert V., Mbananga N. (2002). Obesity in South Africa: The South African demographic and health survey. Obes. Res..

[18] Kautzky-Willer A., Harreiter J., Pacini G. (2016). Sex and gender differences in risk, pathophysiology and complications of type 2 diabetes mellitus. Endocr. Rev..

[19] Mayosi B.M., Lawn J.E., van Niekerk A., Bradshaw D., Abdool Karim S.S., Coovadia H.M. (2012). Health in South Africa: Changes and challenges since 2009. Lancet.

[20] Malakoane B., Heunis J., Chikobvu P., Kigozi N., Kruger W. (2020). Public health system challenges in the Free State, South Africa: A situation appraisal to inform health system strengthening. BMC Health Serv. Res..

[21] Sifunda S., Mbewu A.D., Mabaso M., Manyaapelo T., Sewpaul R., Morgan J.W., Harriman N.W., Williams D.R., Reddy S.P. (2023). Prevalence and Psychosocial Correlates of Diabetes Mellitus in South Africa: Results from the South African National Health and Nutrition Examination Survey (SANHANES-1). Int. J. Environ. Res. Public Health.

[22] Sidahmed S., Geyer S., Beller J. (2023). Socioeconomic inequalities in diabetes prevalence: The case of South Africa between 2003 and 2016. BMC Public Health.

[23] Popkin B.M. (2015). Nutrition Transition and the Global Diabetes Epidemic. Curr. Diab. Rep..

[24] Vorster H.H., Kruger A., Wentzel-Viljoen E., Kruger H.S., Margetts B.M. (2014). Added sugar intake in South Africa: Findings from the Adult Prospective Urban and Rural Epidemiology cohort study. Am. J. Clin. Nutr..

[25] McIlrath L., Slabbert T. (2003). Sedibeng Economic Regeneration Summit.

[26] Sekatane M.B. (2013). An application of different methodologies for measuring poverty in Sharpeville township. Mediterr. J. Soc. Sci..

[27] Oldewage-Theron W.H., Salami L., Zotor F.B., Venter C.S. (2008). Health status of an elderly population in Sharpeville, South Africa. Health SA Gesondheid.

[28] Gibson R.S. (2005). Principles of Nutritional Assessment.

[29] Langenhoven M.L., Kruger M.L., Gouws E., Faber M. (1991). Food Composition Tables.

[30] Ruel M.T. (2003). Operationalizing dietary diversity: A review of measurement issues and research priorities. J. Nutr..

[31] Hatloy A., Torheim L.E., Oshaugh A. (1998). Food variety a good indicator of nutritional adequacy of the diet? A case study from an urban area in Mali. Eur. J. Clin. Nutr..

[32] Krebs-Smith S., Smiciklas-Wright H., Guthrie H., Krebs-Smith J. (1987). The effects of variety in food choices on dietary quality. J. Am. Diet. Assoc..

[33] Matla M.T.H. (2008). The Contribution of Food Access Strategies to Dietary Diversity of Farmworker Households on Oranje Farm in the Fouriesburg District (RSA). Masters’ Dissertation.

[34] Centers for Disease Control and Prevention (CDC) (2007). National Health and Nutrition Examination Survey (NHANES) Anthropometric Procedures Manual.

[35] Burtis C.A., Ashwood E.R., Fowler A.M. (2008). Tietz Fundamentals of Clinical Chemistry.

[36] Shenzhen New Industries Biomedical Engineering Co. Ltd. (2012). Insulin FA (CLIA): Manufacture, 130205002M-v1.0-EN.

[37] Milanović Z., Pantelić S., Trajković N., Sporiš G., Kostić R., James N. (2013). Age-related decrease in physical activity and functional fitness among elderly men and women. Clin. Interv. Aging.

[38] McMurray R.G., Soares J., Caspersen C.J., McCurdy T. (2014). Examining variations of resting metabolic rate of adults: A public health perspective. Med. Sci. Sports Exerc..

[39] Parker E.A., Perez W.J., Phipps B., Ryan A.S., Prior S.J., Katzel L., Serra M.C., Addison O. (2022). Dietary Quality and Perceived Barriers to Weight Loss among Older Overweight Veterans with Dysmobility. Int. J. Environ. Res. Public Health.

[40] Chau D., Cho L.M., Jani P., St. Jeor S.T. (2008). Individualizing recommendations for weight management in the elderly. Curr. Opin. Clin. Nutr. Metab. Care.

[41] Raatz S.K., Conrad Z., Johnson L.K., Picklo M.J., Jahns L. (2017). Relationship of the reported intakes of fat and fatty acids to body weight in US adults. Nutrients.

[42] Beulen Y., Martínez-González M.A., van de Rest O., Salas-Salvadó J., Sorlí J.V., Gómez-Gracia E., Fiol M., Estruch R., Santos-Lozano J.M., Schröder H. (2018). Quality of dietary fat intake and body weight and obesity in a mediterranean population: Secondary analyses within the PREDIMED trial. Nutrients.

[43] Albar S.A. (2022). Dietary Omega-6/Omega-3 Polyunsaturated fatty acid (PUFA) and omega-3 are associated with general and abdominal obesity in adults: UK national diet and nutritional survey. Cureus.

[44] Wang L., Manson J.E., Rautiainen S., Gaziano J.M., Buring J.E., Tsai M.Y., Sesso H.D. (2016). A prospective study of erythrocyte polyunsaturated fatty acid, weight gain, and risk of becoming overweight or obese in middle-aged and older women. Eur. J. Nutr..

[45] Koh-Banerjee P., Chu N.F., Spiegelman D., Rosner B., Colditz G., Willett W., Rimm E. (2003). Prospective study of the association of changes in dietary intake, physical activity, alcohol consumption, and smoking with 9-y gain in waist circumference among 16 587 US men. Am. J. Clin. Nutr..

[46] Bendsen N.T., Chabanova E., Thomsen H.S., Larsen T.M., Newman J.W., Stender S., Dyerberg J., Haugaard S.B., Astrup A. (2011). Effect of trans fatty acid intake on abdominal and liver fat deposition and blood lipids: A randomized trial in overweight postmenopausal women. Nutr. Diabetes.

[47] Scholz A., Navarrete-Muñoz E.M., García-de-la-Hera M., Fernandez-Somoano A., Tardon A., Santa-Marina L., Pereda-Pereda E., Romaguera D., Guxens M., Beneito A. (2019). Association between Trans Fatty Acid Intake and Overweight Including Obesity in 4 to 5-year-old Children from the INMA Study. Pediatr. Obes..

[48] Sun J., Ruan Y., Xu N., Wu P., Lin N., Yuan K., An S., Kang P., Li S., Huang Q. (2023). The effect of dietary carbohydrate and calorie restriction on weight and metabolic health in overweight/obese individuals: A multi-center randomized controlled trial. BMC Med..

[49] Bazzano L.A., Hu T., Reynolds K., Yao L., Bunol C., Liu Y., Chen C.S., Klag M.J., Whelton P.K., He J. (2014). Effects of low-carbohydrate and low-fat diets: A randomized trial. Ann. Intern. Med..

[50] Suara S.B., Siassi F., Saaka M., Foroshani A.R., Asadi S., Sotoudeh G. (2020). Dietary fat quantity and quality in relation to general and abdominal obesity in women: A cross-sectional study from Ghana. Lipids Health Dis..

[51] Torres-Castillo N., Silva-Gómez J.A., Campos-Perez W., Barron-Cabrera E., Hernandez-Cañaveral I., Garcia-Cazarin M., Marquez-Sandoval Y., Gonzalez-Becerra K., Barron-Gallardo C., Martinez-Lopez E. (2018). High dietary ω-6:ω-3 PUFA ratio is positively associated with excessive adiposity and waist circumference. Obes. Facts.

[52] Oldewage-Theron W., Egal A.A. (2021). Is food insecurity a problem among the elderly in Sharpeville, South Africa?. Food Secur..

[53] United Nations Children’ Emergency Fund (1990). Conceptual Framework of Undernutrition.

[54] Opperman M., Marais D.W., Spinnler Benade A.J. (2011). Analysis of omega-3 fatty acid content of South African fish oil supplements. Cardiovasc. J. Afr..

[55] Evert A.B., Boucher J.L., Cypress M., Dunbar S.A., Franz M.J., Mayer-Davis E.J., Neumiller J.J., Nwankwo R., Verdi C.L., Urbanski P. (2013). Nutrition therapy recommendations for the management of adults with diabetes. Diabetes Care.

[56] Due A., Larsen T.M., Hermansen K., Stender S., Holst J.J., Toubro S., Martinussen T., Astrup A. (2008). Comparison of the effects on insulin resistance and glucose tolerance of 6-mo high-monounsaturated-fat, low-fat, and control diets. Am. J. Clin. Nutr..

[57] Vasishta S., Ganesh K., Umakanth S., Joshi M.B. (2022). Ethnic disparities attributed to the manifestation in and response to type 2 diabetes: Insights from metabolomics. Metabolomics Off. J. Metabolomic Soc..

[58] Weir N.L., Nomura S.O., Steffen B.T., Guan W., Karger A.B., Klein R., Klein B.E.K., Cotch M.F., Tsai M.Y. (2020). Associations between omega-6 polyunsaturated fatty acids, hyperinsulinemia and incident diabetes by race/ethnicity: The Multi-Ethnic Study of Atherosclerosis. Clin. Nutr..

[59] Baghdasarian S., Lin H.P., Pickering R.T., Mott M.M., Singer M.R., Bradlee M.L., Moore L.L. (2018). Dietary cholesterol intake is not associated with risk of type 2 diabetes in the Framingham offspring study. Nutrients.

[60] Collino M., Aragno M., Castiglia S., Miglio G., Tomasinelli C., Boccuzzi G., Thiemermann C., Fantozzi R. (2010). Pioglitazone improves lipid and insulin levels in overweight rats on a high cholesterol and fructose diet by decreasing hepatic inflammation. Br. J. Pharmacol..

[61] Basciano H., Miller A.E., Naples M., Baker C., Kohen R., Xu E., Su Q., Allister E.M., Wheeler M.B., Adeli K. (2009). Metabolic effects of dietary cholesterol in an animal model of insulin resistance and hepatic steatosis. Am. J. Physiol. Endocrinol. Metab..

